# Poloxamer‐Enabled Thermoresponsive Biobased Controlled‐Release Fertilizer for On‐Demand Nutrient Delivery

**DOI:** 10.1002/advs.78371

**Published:** 2026-10-18

**Authors:** Jiahui Liu, Junyin Li, Ziyao Li, Xiangjie Zhao, Fengyan Fang, Yan Liu, Songyuan Wang, Boning Su, Yuanyuan Yao, Bin Gao, Shugang Zhang, Yuechao Yang

**Affiliations:** ^1^ National Engineering Research Center for Efficient Utilization of Soil and Fertilizer Resources National Engineering & Technology Research Center for Slow and Controlled Release Fertilizers College of Resources and Environment Shandong Agricultural University Taian Shandong China; ^2^ Department of Civil and Environmental Engineering Rensselaer Polytechnic Institute Troy New York USA; ^3^ State Key Laboratory of Wheat Improvement Shandong Agricultural University Taian China; ^4^ Department of Soil and Water Sciences Tropical Research and Education Center Institute of Food and Agricultural Sciences University of Florida Homestead Florida USA

**Keywords:** intelligent controlled release, precise fertilization, sustainable agriculture, temperature response

## Abstract

Synchronizing nutrient release of fertilizers with crop uptake remains a central challenge in precision agriculture. Here, we present a novel temperature‐responsive biobased controlled‐release fertilizer (TRBFs) incorporating Poloxamer 407 (F127) as a thermal switch within a castor oil‐derived polyurethane coating. The optimized TRBF10 (3 wt.% coating rate, 10 wt.% F127 in coating) exhibits a sharp, reversible phase transition at ∼20°C, matching the phenological requirements of overwintering crops. As quantified by digital refractometry, the 30‐day cumulative nitrogen release is suppressed to 9.22% at 10°C (crop dormancy) and increases 8.7‐fold to 80.32% at 30°C (active growth). This scalable technology overcomes intrinsic limitations of conventional diffusion‐controlled coated fertilizers and offers potential for on‐demand nutrient delivery with reduced environmental loss. Our work establishes sustainable strategies for climate‐adaptive precision agriculture and offers new insights and design paradigms for intelligent controlled‐release materials.

## Graphical abstract

A bio‑based thermoresponsive controlled‑release fertilizer (TRBF) is fabricated by introducing F127 into a castor‑oil‑derived polyurethane coating. Distinct from conventional bio‑based coated fertilizer (BCF), TRBF achieves temperature‑regulated nitrogen release via F127 reversible phase transition, suppressing nutrient leakage at low temperature and accelerating nitrogen supply under high temperature for climate‑adaptive crop nutrition.

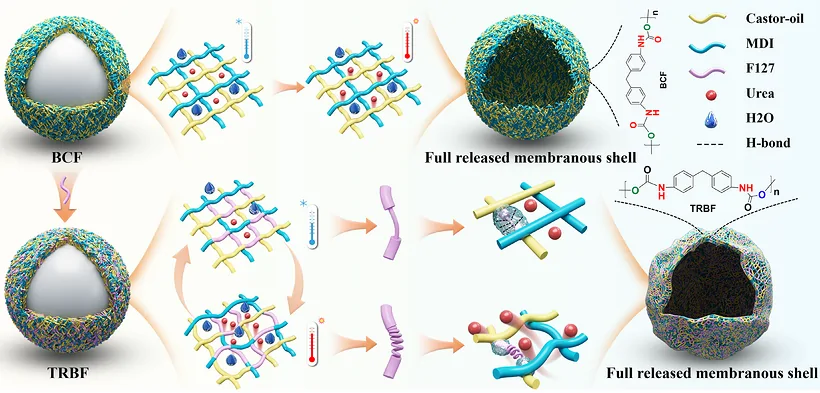

## 1 Introduction

In the context of global sustainable agricultural development [1, 2, 3], global food demand, climate‐driven shifts in crop nutrient demand, and nitrogen losses create formidable challenges for crop nutrient management [4, 5, 6]. These challenges have driven advances in smart controlled‐release fertilizers that enable on‐demand nutrient delivery while reducing environmental impact. Controlled‐release fertilizers (CRFs) can increase fertilizer utilization efficiency [7] and reduce nutrient loss [8], contributing to sustainable agricultural production [9, 10, 11]. However, current research on CRFs focuses almost exclusively on extending release longevity [12, 13] while neglecting the precise coordination between nutrient release and crop demand.

Nutrient uptake by crops is closely related to various environmental factors [14, 15], among which temperature plays a critical regulatory role [16, 17, 18, 19]. Temperatures above 20°C typically promote crop metabolic activity, accelerating nutrient uptake and translocation. In contrast, temperatures below 10°C slow or even inhibit crop growth, markedly reducing nitrogen demand and root uptake rates [20, 21, 22, 23]. Therefore, regulating nutrient release according to temperature to synchronize it with crop fertilizer demand and growth is a key strategy for achieving precision agriculture. This is especially true for winter wheat, which undergoes a 6‐month cycle with significant temperature fluctuations during its growth period. For example, winter wheat experiences high nutrient demand during the jointing‐to‐flowering stage [24, 25, 26, 27], when the average surface soil temperature is typically around 15°C. However, the nutrient release rate of CRFs is generally lower than crop demand at this temperature. Thus, developing a CRF that can intelligently regulate nutrient release in response to temperature changes is highly important for improving nutrient utilization efficiency and increasing the yield of winter wheat [28, 29].

A key solution to the above problems is the development of temperature‐responsive intelligent coating materials [30, 31]. Research on thermosensitive materials has provided a solid foundation [32, 33, 34], and these materials are widely applied in fields such as food packaging and medicine [35, 36, 37]; however, research on their application in agriculture remains limited. Qiao et al. developed a PPG/PCL2000 blend polyurethane‐coated temperature‐responsive CRF (4 wt.% coating rate, PPG/PCL molar ratio = 8:2) [38]. Its hourly nitrogen release rate increased by 22.3‐fold at 33°C, but this high response threshold and ∼30‐day release longevity are unsuitable for most overwintering field crops. Shang et al. fabricated a CMC‐stabilized N‐vinylcaprolactam thermoresponsive hydrogel (max swelling ratio = 2056%) [39]. Despite good water retention, its nutrient loading is far lower than granular coated fertilizers, and 80% of urea is released within 12 h at 25°C, showing poor long‐term low‐temperature controlled release. In summary, the development of temperature‐responsive CRFs remains a significant gap in the current research field.

In this work, biobased materials (e.g., starch [40], cellulose [41], biochar [42], and epoxy resins [43]) with renewability and environmental friendliness [44, 45] are used as the base of thermosensitive materials to coat urea [46, 47] for the development of temperature‐responsive CRFs. Castor oil (CO), as a bioresource, has received extensive research attention because of its abundant hydroxyl groups and excellent biodegradability [48, 49, 50]. Poloxamer 407 (F127) is a nonionic surfactant with good biocompatibility and thermal sensitivity [51, 52], and it has different hydration states at different temperatures [53, 54]. Here, the incorporation of F127 into the CO‐MDI polyurethane system for temperature‐responsive coating materials is reported for the first time. Based on this innovation, temperature‐responsive biobased fertilizers (TRBFs) with 3 wt.% coating rates were prepared through a drum coating process. We systematically screened F127 loadings (5–20 wt.%) to identify the optimal formulation, characterized the corresponding nitrogen release profiles across −4°C to 35°C, and elucidated the associated release kinetic models. This innovative and precise release mechanism of TRBFs overcomes the traditional fixed‐release‐rate mode and represents a new research pathway that precisely aligns with plant nutrient uptake patterns. It offers a highly promising direction for the future development of CRFs and guides the fertilizer industry toward precision, efficiency, and intelligence.

## 2 Materials and Methods

### 2.1 Materials

Urea (diameter: 2–4 mm, 46.4% N, purchased from Shandong Agricultural University Fertilizer Technology Co., Ltd.), Castor oil (CP, Tianjin Komio Chemical Reagent Co., Ltd.), diphenylmethane diisocyanate (MDI, 30.03 wt.% ‐NCO group, Yantai Wanhua Chemical Group Co., Ltd.), poloxamer 407 (Pluronic F‐127, Shanghai Yuanye Biotechnology Co., Ltd.), and acetone (AR, Tianjin KaiTong Chemical Reagent Co., Ltd.).

### 2.2 Preparation of Polyurethane Coatings

F127 was weighed and placed into a small plastic cup with a small amount of acetone, after which the CO and MDI were added. The raw materials were mixed evenly, poured into a Teflon plate mold, and then placed in a 50°C vacuum drying oven to remove bubbles. After 10 min, the sample was removed to eliminate the bubbles. This step was repeated three times to remove as many bubbles as possible during the reaction process. Finally, the mold was transferred to a 50°C oven for curing to obtain the solidified product.

### 2.3 Preparation of Various Coated CRFs

#### 2.3.1 Preparation of BCF

Following the conventional drum‐coating method, a one‐step procedure was adopted to prepare a biobased CRF (BCF). Urea granules were loaded into a 50°C rotating drum (Model WKY‐300; Qingzhou Jingcheng Medical Instrument Co., Ltd.), and the rotary speed was set to 27 rpm. The CO, MDI, and acetone were homogenized in a small plastic cup and then slowly poured onto the tumbling urea surface. Each coating cycle employed 0.5 wt. % of the total urea mass. After six consecutive cycles, BCF with a final coating rate of 3% was obtained. Complete mass ratio values and experimental parameters of all samples can be found in Table S1.

#### 2.3.2 Preparation of TRBFs

F127 was weighed and added to a plastic cup with a small amount of acetone (covering F127), after which the mixture was gently shaken until it was slightly soluble. Subsequently, the CO and MDI were introduced, and the blend was stirred uniformly to obtain the coating precursor for TRBFs. The coating materials were applied onto the surface of the fertilizer, and the coating procedure for BCF was repeated as described above, resulting in a coating thickness of 3%. TRBFs containing 5%, 10%, and 20% F127 (by weight of total coating material) were designated TRBF5, TRBF10, and TRBF20, respectively (Figure 1). The molecular structure and synthesis mechanism are shown in Figure S1.

**FIGURE 1:**
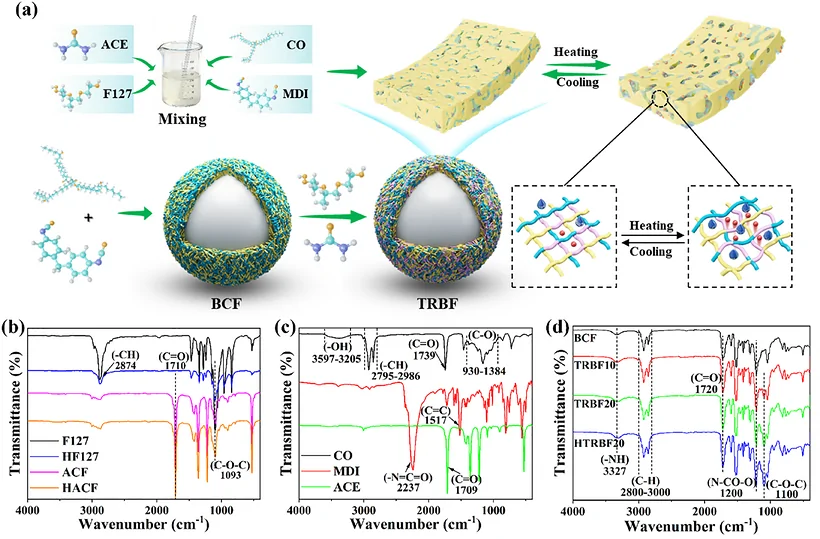
(a) Schematic of the synthesis. FTIR spectra of (b) F127 in different states, (c) raw materials, and (d) the as‐prepared CRFs.

#### 2.3.3 Preparation of HTRBF

The F127 and CO were weighed into a small plastic cup and placed in a 60°C oven. After 15 min, the solids were completely molten and mixed evenly with MDI, and the final product was slowly poured into the coating drum. The addition amount of F127 was 20%, and the coating thickness was 3%. The resulting fertilizer was designated HTRBF20.

#### 2.3.4 Preparation of DTRBF

The preparation of DTRBF was carried out by the “4+2” method for coating. First, four successive layers were applied using the BCF formulation, followed by two additional layers prepared with the TRBF20 formulation. The resulting double‐layer CRFs (DTRBF20) possessed a 2% BCF inner layer and a 1% TRBF20 outer layer.

### 2.4 Characterization

The structural properties of the raw materials and products were analyzed using FTIR (Bruker TENSOR II). The surface morphology of the products was observed using SEM (GeminiSEM 300), EDX, and AFM (German Bruker Dimension ICON). The thermal stability of the membrane shell was investigated using TGA (Shimadzu, Japan) with a heating rate of 10°C/min and a temperature range of 30°C–800°C. Combined with py‐GCMS (GCMS‐QP2020NX), the chemical composition and structural information of the membrane shell after high‐temperature cracking were explored. DSC (Swiss Mettler DSC3) and XRD (Japanese Rigaku SmartLab SE) were used to investigate the phase transition temperature and crystal structure of the membrane shell. The densities (American Mc AccuPyc II 1340) of different polyurethanes were also tested. Liquid nuclear magnetic resonance (NMR) and solid‐state nuclear magnetic resonance (solid‐state NMR) were employed to investigate the microscopic dynamic changes in F127 in response to temperature. In situ FTIR (German Bruker VERTEX 80v) and XPS (Thermo Scientific K‐Alpha) were utilized to explore the structural changes in the membrane shell at different temperatures. Dynamic scanning mode of DMA (Netzsch DMA242E) was used to test the viscoelastic properties of the polyurethane films at different temperatures, with a test temperature range of −50°C to 350°C, a test frequency of 1 Hz, and a heating rate of 5°C/min. The pore volume changes of BCF and TRBF at different temperatures were quantitatively analyzed using low‐field NMR (LF‐NMR, Nymai MesoMR12‐060H‐I) testing (the sample was 100% saturated with water, the temperature was corrected, and water quality‐signal linear calibration was adopted). The water vapor permeability (abbreviated as WVP, W3/031) was measured to reflect the changes in pore size and permeability of the polyurethane film at different temperatures. Molecular dynamics (MD) simulations were performed to reveal the molecular mechanism responsible for the temperature responsiveness of the coating.

### 2.5 Determination of N Release Characteristics

The determination of CNRs and stage nitrogen release rates (SNRs) was conducted by a refractive index analysis method. A standard curve series was prepared by dissolving 0, 0.5, 1, 2, 5, 8, and 10 g of CRF in 200 mL of deionized water, corresponding to 0%, 5%, 10%, 20%, 50%, 80%, and 100% of the theoretical maximum release, respectively. Each concentration was measured in triplicate using a digital refractometer (Atago PAL‐α, ±0.0001 nD). A linear relationship between the urea concentration (C, % release) and refractive index (n) was established:

$$n=6.8833C+250.8\left(R^{2}=0.9999,n=6\right)$$

Method validation demonstrated good precision (RSD: 0.05%–0.16%, n = 6) and accuracy (spike recovery: 100.73%–102.02%). This equation was subsequently used to convert the measured RI values into percentages of released nitrogen.

Following the established calibration equation, nitrogen release profiles were obtained for BCF, TRBF5, TRBF10, TRBF20, HTRBF20, and DTRBF20. Ten grams of each coated fertilizer sample were sealed in a small nylon mesh pouch and immersed in 200 mL of deionized water inside a hermetic plastic bottle. Afterward, the bottles were incubated at the required temperatures (‐4°C, 4°C, 10°C, 15°C, 20°C, 25°C, 30°C, and 35°C). On predetermined sampling days, 5 mL aliquots were withdrawn for refractive index measurements. After each sampling, the remaining solution was discarded, the pouch was gently blotted to remove excess water, and 200 mL of fresh deionized water was added to resume incubation. All the treatments were performed in triplicate.

## 3 Results and Discussion

### 3.1 FTIR Results of Raw and Coating Materials

FTIR spectroscopy was employed to investigate the structural changes of the raw materials and the products. The characteristic peaks of F127 were primarily C─H (2874 cm^−1^) and C─O─C (1093 cm^−1^). After being heated to 65°C (HF127), it transformed from a powder state to a gel state, but there was no obvious change in the C─O─C absorption peaks. This finding indicates that the basic structure of F127 remains unchanged and the heat‐induced transformation is a physical self‐assembly process. Compared to F127, the intensity of the C─H vibrational peaks of HF127 significantly decreased, probably because of enhanced chain segment motion at high temperature and the hydrophobic aggregation of its hydrophobic portion of F127, which formed a micellar core and restricted the symmetric stretching vibrational freedom of the methyl groups. When F127 was added to acetone at room temperature (ACF in Figure 1b) and at 65°C (HACF in Figure 1b), the FTIR spectra showed no significant changes except for the appearance of C═O at 1710 cm^−1^, which is the characteristic peak of acetone (ACE in Figure 1c). This suggests that the addition of acetone did not affect the chemical structure of F127.

The FTIR spectra of the other coating raw materials were also determined. The absorption peaks of CO are ─OH (3597–3205 cm^−1^), ─CH (2795–2986 cm^−1^), C═O (1739 cm^−1^), and C─O (930–1384 cm^−1^). The absorption peak of MDI is N═C═O (2237 cm^−1^), and the stretching vibration of the benzene ring is C═C (1517 cm^−1^) (Figure 1c). Upon completion of the reaction to produce polyurethane, the carbamate bond (1200 cm^−1^) is formed, the ─OH is completely consumed, and the residual isocyanate group forms a single and wide absorption peak at 3327 cm^−1^ (Figure 1d). Notably, TRBF10, TRBF20, and HTRBF20 all contain the ether bond C─O─C of F127 (1100 cm^−1^), whereas BCF does not have this characteristic peak. This proves that F127 is involved in the formation of reaction products and is successfully embedded in the molecular structure of the coating materials.

### 3.2 Surface Morphology of the CRF Coating Shells

The coated urea granules are shown in Figure 2a,b. The water contact angle of the BCF membrane is slightly higher than that of the TRBF membrane. The element distribution and surface morphology of BCF and TRBF10 coating shells were observed by EDX, SEM, and AFM. These coatings mainly contained three elements, C, N, and O, which are uniformly distributed. The C content of TRBF10 (66.31%) was slightly lower than that of BCF (71.29%), mainly because part of the CO used in the raw material was replaced by F127 during TRBF preparation. The two coatings were measured at 12.28 and 14.22 µm, respectively, indicating their ultrathin nature (Figure 2c,d).

**FIGURE 2:**
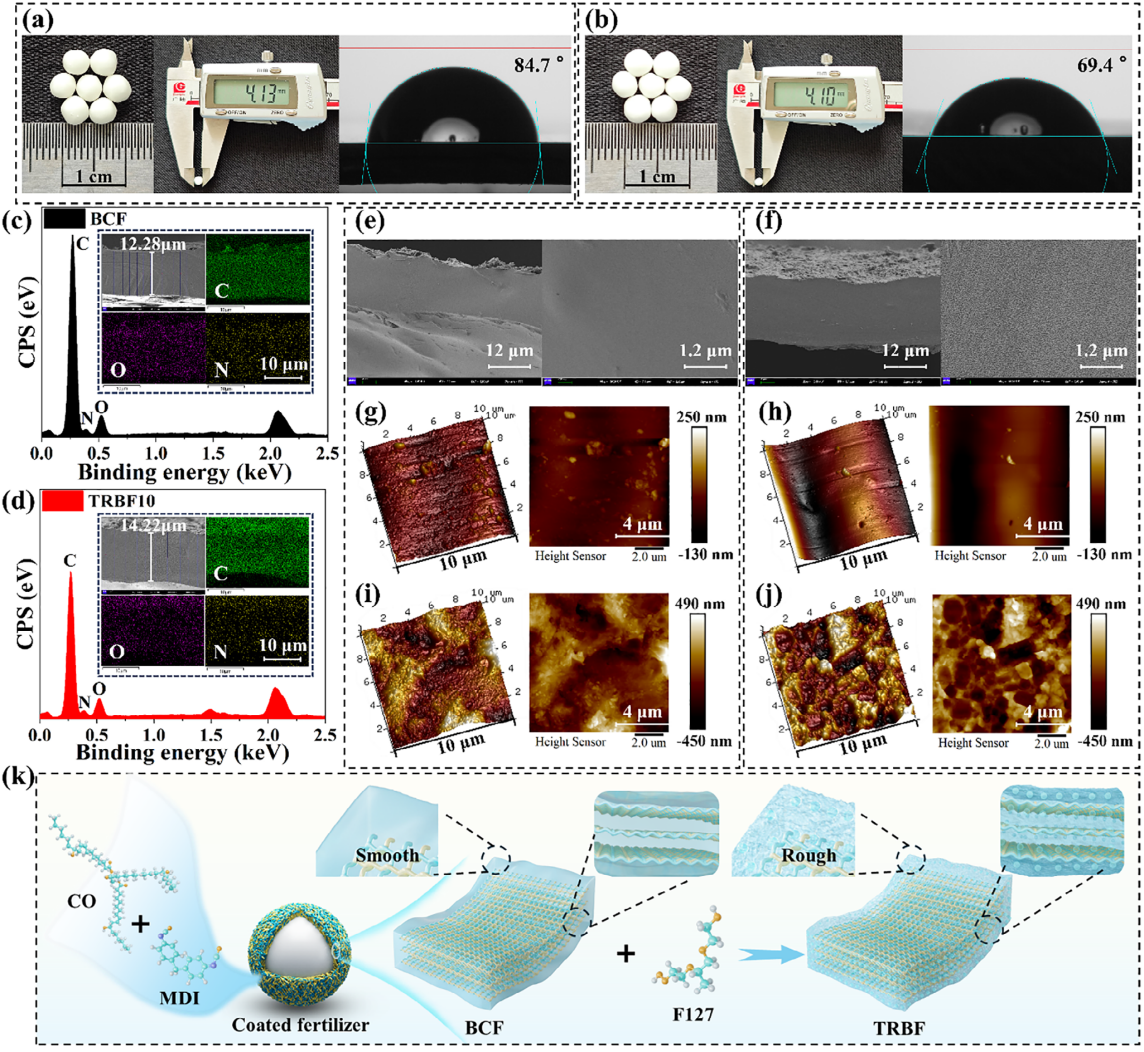
Digital photographs, diameters and water contact angles of (a) BCF and (b) TRBF10. EDX spectra, EDX maps, and membrane shell thickness measurement chart of (c) BCF and (d) TRBF10. SEM images of the fracture surface with 2000 × magnification and 20 000 × magnification from (e) BCF and (f) TRBF10. AFM images of (g) outer and (i) inner BCF. AFM images of (h) outer and (j) inner regions of TRBF10. (k) Surface schematic of BCF and TRBF.

The outer surfaces of BCF and TRBF10 were smooth and flat, thus having good film‐forming properties. After the complete release of nutrients, many tears and pores formed on their inner surface (Figure S2), but these defects were confined to the surface layer and did not damage the internal structure of the coatings. At a magnification of 20 000×, the cross section of BCF remained smooth and flat, whereas the cross section of TRBF showed a dense and uniformly distributed granular structure. This indicates that F127 may have changed the surface homogeneity of the reaction product by reducing the surface energy or regulating the kinetics.

AFM was also used to study the micromorphology and roughness of BCF and TRBF (Figure 2g–j). The outer surface roughness of BCF and TRBF10 was 29.5 and 65.8 nm, respectively, and the inner surface roughness was 139 and 173 nm, respectively. The roughness of TRBF was slightly greater than that of BCF, indicating that the addition of F127 increased the surface roughness of the coating material, which is consistent with the SEM results. The alternating light and dark contrast were clearly observed in the AFM images of the inner surface of the TRBF, forming uniformly distributed dark patches (Figure 2j). This suggests that the addition of F127 may have altered the phase separation behavior during the reaction process, thereby promoting particle formation or aggregation to increase coating roughness.

### 3.3 Integrated Thermal and Structural Characterization

The thermal stability of F127 and the coatings were analyzed using TGA and DTG (Figure 3a,b). BCF exhibited three main stages of mass loss. The first stage (221°C–343°C) is dominated by the breaking of carbamate bonds. The second stage (343°C–416°C) is dominated by the decomposition of soft segments and organic molecular chains. The third stage (416°C–570°C) is the dehydration of hydroxyl groups and the decomposition of polyol residues [55, 56, 57]. F127 was relatively stable below 170°C, and the weight decreased rapidly at 170°C–420°C. During this stage, polyoxyethylene‐polyoxypropylene decomposes with cleavage of the ether bond (C‐O‐C), ultimately leading to its near‐complete degradation. There was no notable changes in the 50% degradation temperature (T_50%_), maximum weight loss rate temperature (T_max_), or residual mass, indicating that the addition of a small amount of F127 did not affect the thermal stability of polyurethane.

**FIGURE 3:**
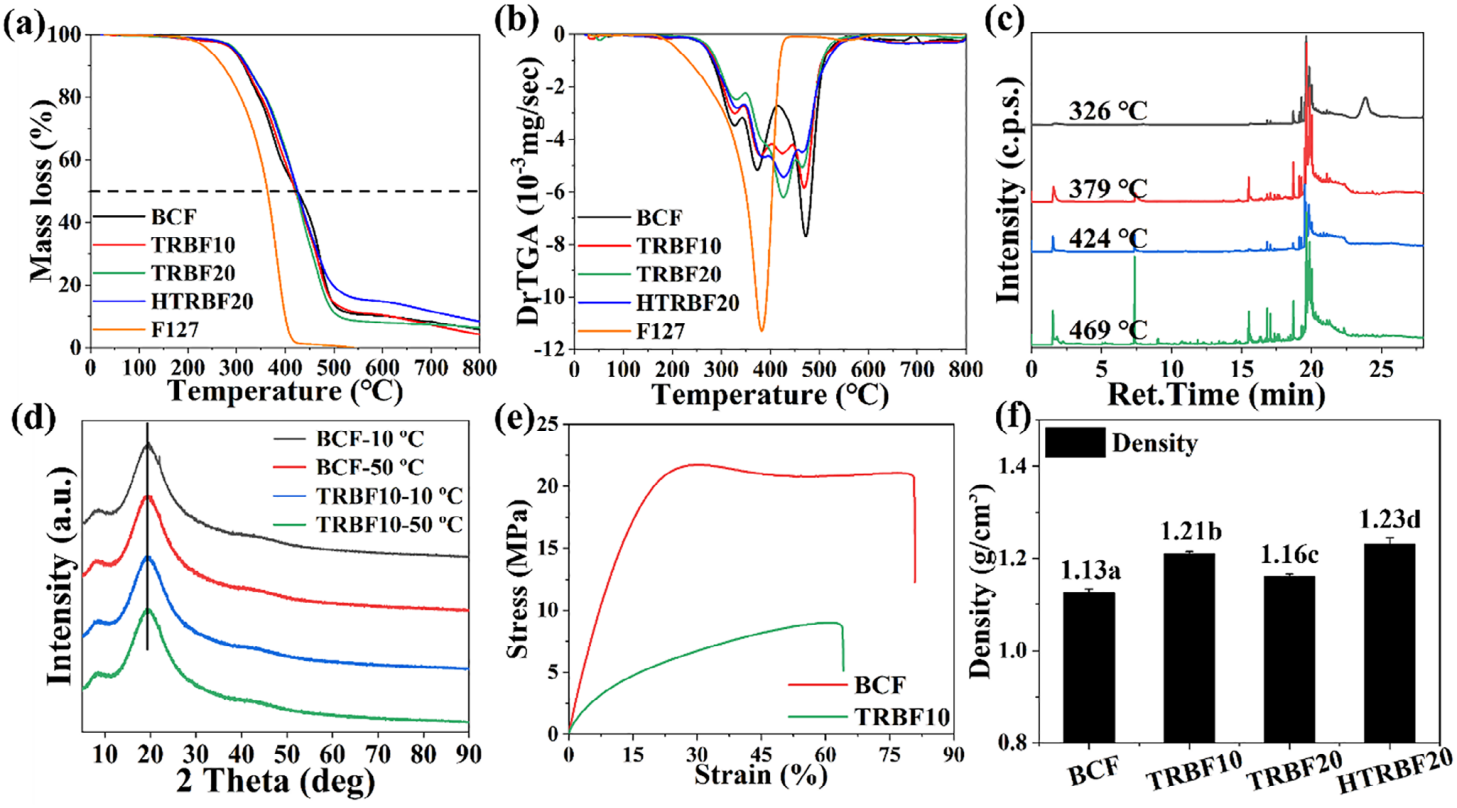
(a) TGA and (b) DTG curves of BCF, TRBF10, TRBF20, HTRBF20, and F127. (c) Py‐GCMS spectrum of TRBF10. (d) XRD curves of BCF and TRBF10 at 10°C and 50°C. (e) Stress–strain curves of BCF and TRBF10. (f) Density patterns of BCF, TRBF10, TRBF20, and HTRBF20. All data are presented as mean ± standard deviation (*n* = 5). Statistical differences were evaluated by one‐way ANOVA followed by Tukey's HSD post‐hoc test. Different lowercase letters above bars indicate significant differences between groups (*p*< 0.05).

Compared with the BCF treatment, the TRBF and HTRBF treatments showed four DTG peaks, with an additional mass‐loss stage attributable to the addition of F127. To explore the product composition and structure of the decomposition process of TRBF coatings, py‐GCMS (Figure 3c) was conducted on TRBF10, with the maximum peaks of each phase of the DTG as the test temperature points (326°C, 379°C, 424°C, and 469°C). The first mass loss stage of TRBF10 (227°C–347°C) exhibited 15 peaks, and the remaining components mainly consisted of benzene rings, oleic acids, etc. Therefore, this phase is attributed to carbamate bond decomposition. In the second stage (347°C–400°C, 30 peaks), small amounts of aldehyde and ether compounds were produced. In combination with the DTG curve of F127, the generation of these substances can be related to the oxidation and thermal decomposition of the polyether chain segments. The third stage (400°C–451°C, 31 peaks) primarily involved the decomposition of polyols, resulting in the formation of many ester compounds. However, owing to the addition of F127, the temperature at which this stage occurred was elevated. The fourth stage (451°C–548°C) showed 41 peaks, which correspond to the further decomposition and oxidation of residual carbides, resulting in the production of a large number of small‐molecule esters, alcohols, and other compounds.

XRD was also used to investigate the crystal structure of the TRBFs (Figure 3d). Additionally, density and Stress‐strain curves were measured to further characterize their structural characteristics (Figure 3e,f). The XRD curves of all the treatments exhibited a strong single diffraction peak at approximately 2θ = 19.22°. At different temperatures (4°C and 50°C), the peak values and positions of all the materials did not change, indicating that temperature variations did not affect the crystal structure of the materials. The BCF and TRBF10 membranes were stretched at a speed of 10 mm/min to evaluate the stretching performance. The maximum tensile strengths of BCF and TRBF10 were 21.73 and 9.02 MPa, respectively, and the corresponding maximum strains were 80.74% and 64.02%, respectively. The Young's modulus of BCF was 153.69 MPa, while that of TRBF10 was 52.16 MPa. There was no significant difference in density between BCF and TRBFs. Significant bulk‐density differences existed across four fertilizer samples (*p*< 0.001). BCF had the lowest density (1.1251 ± 0.0089 g/cm^3^), TRBF20 showed intermediate values (1.1604 ± 0.0059 g/cm^3^), while TRBF10 and HTRBF20 yielded higher densities of 1.2095 ± 0.0054 g/cm^−3^ and 1.2305 ± 0.0144 g/cm^3^, respectively. Notably, the Young's modulus, tensile strength, and elongation at break of BCF were all higher than those of TRBF10, indicating that BCF possesses superior overall rigidity and toughness compared to TRBF10. This phenomenon can be attributed to the more abundant physical interactions in BCF, which can effectively enhance the resistance to elastic deformation.

### 3.4 Nutrient Release Rate in Response to Temperature

To investigate the effect of the F127 modifier on the controlled‐release performance of the CRFs, the nutrient release characteristics of BCF, TRBF5, TRBF10, TRBF20, HTRBF20 and DTRBF20 at different temperatures (−4°C, 4°C, 10°C, 15°C, 20°C, 25°C, 30°C, and 35°C) were evaluated (Figure 4a–d and Figure S3). Physical photos of fertilizer cultivation and SNR diagrams are shown in Figures S3 and S4. At −4°C, the CNRs of BCF, TRBF5 and TRBF10 at 63 d were 10.07% ± 0.1%, 3.00% ± 0.08% and 3.97% ± 0.1%, respectively. At 4°C, their CNRs were 16.17% ± 0.13%, 5.23% ± 0.1%, and 9.01% ± 0.1%, respectively. The CNRs in the TRBF treatments were much lower than those of BCF, indicating that the addition of F127 suppressed nutrient release under low‐temperature conditions. At 10°C and 15°C, the CNRs of BCF, TRBF5, and TRBF10 at 63 d were 20.44% ± 0.48%, 11.23% ± 1.6%, and 18.26% ±0.34% and 20.92% ± 0.13%, 7.65% ± 0.13%, and 16.66% ± 0.15%, respectively. The trend of the CNRs from TRBF10 gradually converged to the BCF with increasing temperature. When the temperature reached 20°C, the CNRs of BCF and TRBF10 were 14.90% ± 0.32% and 14.75% ± 0.17%, respectively, at 21 d; the CNRs of BCF and TRBF5 were 25.70% ± 0.56% and 35.54% ± 1.54%, respectively, at 35 d. Subsequently, the release rate of TRBFs gradually exceeded that of BCF. At 25°C, 30°C, and 35°C, the CNRs of TRBF5 exceeded those of BCF on approximately the 16th, 10th, and 5th days, whereas the CNRs of TRBF10 exceeded those of BCF on approximately the 9th, 6th, and 1st days. These results indicate that appropriate incorporation of F127 can suppress nutrient release at low temperatures while accelerating nutrient release as the ambient temperature increases. However, the temperature‐responsive performance was not monotonically improved by increasing the F127 content. Among the tested formulations, TRBF10 achieved the best balance between low‐temperature nutrient retention and high‐temperature release acceleration, which closely aligns with the dynamic nutrient absorption patterns of crops and provides a more precise nutrient‐supply strategy for plant growth.

**FIGURE 4:**
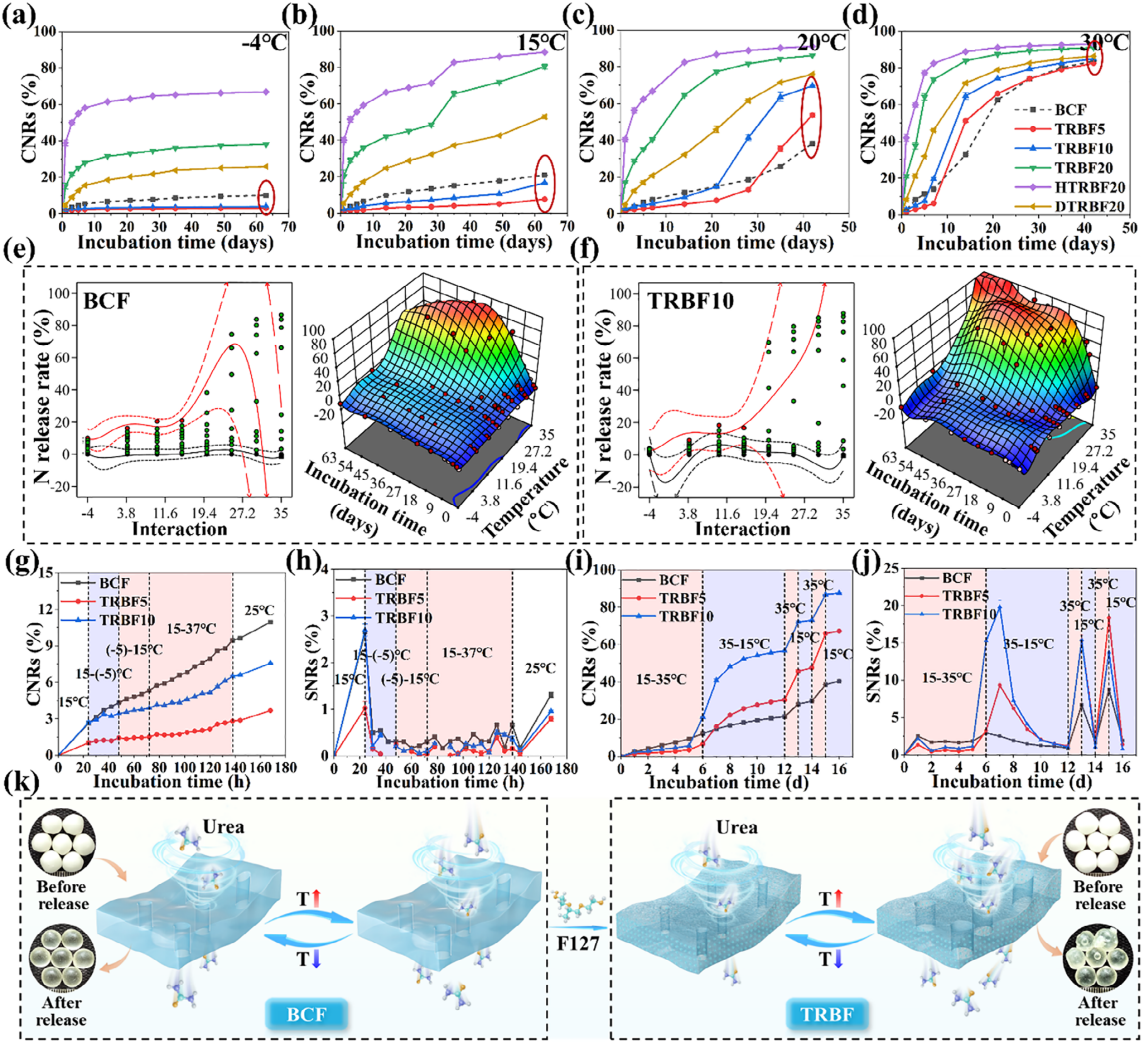
(a–d) Cumulative nitrogen release rates of BCF, TRBF5, TRBF10, TRBF20, HTRBF20, and DTRBF20 at different temperatures. The factor–effect relationship diagram and 3D response surface construction model of (e) BCF and (f) TRBF10. (g,i) Cumulative release rates and (h,j) Stage release rates of BCF, TRBF5, and TRBF10 under dynamic temperature conditions. (k) Schematic diagram of nutrient release. Each data point is the mean of three independent measurements (n = 3). Mean and standard deviation values are provided in the Release Rate Dataset.

When the F127 content increased to 20%, TRBF20 still exhibited temperature‐responsive release behavior, but its overall controlled‐release performance was weakened, especially under low‐temperature conditions. This result suggests that excessive F127 disrupts the stable cross‐linked polyurethane structure, weakens the barrier property of the coating, and creates more water‐accessible domains, leading to undesirably high nutrient release even at low temperatures. The release performance of HTRBF20 was inferior to that of TRBF20, likely because the enhanced mobility of F127 molecular chains induced conformational changes and affected its incorporation into the polyurethane network. DTRBF20, prepared using the “4+2” coating method, showed better controlled‐release performance than TRBF20, further confirming that an optimized F127 distribution and content are essential for maintaining coating integrity. Therefore, TRBF10 was identified as the optimal formulation, whereas excessive F127 loading deteriorated the inherent controlled‐release performance of the coating.

To better understand the effects of TRBF in response to temperature, we constructed a model to investigate the relationships among the number of culture days, release rate, and temperature under different F127 amounts (Figure 4e,f). In the factor–effect relationship diagram, the green dots represent the actual measured values, the red dotted lines represent the confidence intervals, and the red solid lines represent the magnitude of the influence of temperature on the release rate after fitting. In the BCF treatment, the influence of temperature on the release rate was relatively small. When the temperature exceeded 25°C, further increases in temperature resulted in a diminishing effect on the release rate of BCF. In contrast, in the TRBF10 treatment, the influence curve continued to rise with increasing temperature, demonstrating a more pronounced effect of temperature on the release rate. A 3D response surface diagram provides more intuitive visualization of the interrelationships among various factors and enables prediction of nutrient release under different conditions. Compared with that of the BCF treatment without the modifier, the slope of TRBF10 is steeper, indicating that TRBF10 is more sensitive to temperature. The detailed equations for model establishment and other modeling results are shown in Figure S5.

To further evaluate the temperature responsiveness and reversibility of the TRBF, two groups of dynamic temperature‐controlled experiments were conducted. In the first group, the temperature gradually decreased from 15°C to −5°C at a rate of 4°C per 6 h and then increased to 37°C, with nutrient release measured simultaneously (Figure 4g,h). The second group adopted a temperature of 4°C per 24 h and eventually switched repeatedly between 15°C and 35°C (Figure 4i,j). The results demonstrated that when the temperature change cycle was only 6 h, the release kinetics of the TRBFs did not differ significantly from those of the BCF controls, indicating a kinetic lag in the thermal response of the coating. Extending the cycle to 24 h, however, revealed a pronounced temperature dependence: the nutrient release of TRBFs was lower than that of BCF, and TRBFs exhibited suppressed release at low temperatures. In contrast, at higher temperatures, nutrient release of TRBFs was markedly accelerated. This “inhibit‐accelerate” switching was fully reversible across multiple cycles. Such a release pattern can reduce nutrient loss during cold periods and promote nutrient release during periods of high crop nutrient demand, which is driven by the accumulation of temperature.

### 3.5 Temperature Response Mechanism

The F127 solution exhibited temperature‐responsive properties. When the temperature increased to approximately 30°C, it solidified into a gel (Figure 5a). The DSC curve of F127 shows a distinct exothermic peak at approximately 58.86°C, indicating the micellization of F127. At approximately 22.66°C, there was an endothermic peak, indicating the gel dissociation of F127 and the occurrence of a phase transition (Figure 5b). The DSC spectrum of the synthesized polyurethane shows an exothermic peak at approximately 40°C, which might be the crystallization peak of the polyurethane soft segment. The crystallization melting peak of TRBF5 and TRBF20 occurred at approximately 125°C, which can be attributed to the formation of crystalline domains or physical cross‐linking points by F127 in the polyurethane. However, the peak at 125°C for the TRBF10 treatment disappeared after elimination of the thermal history (Figure S6), indicating that TRBF10 is the optimal formula.

**FIGURE 5:**
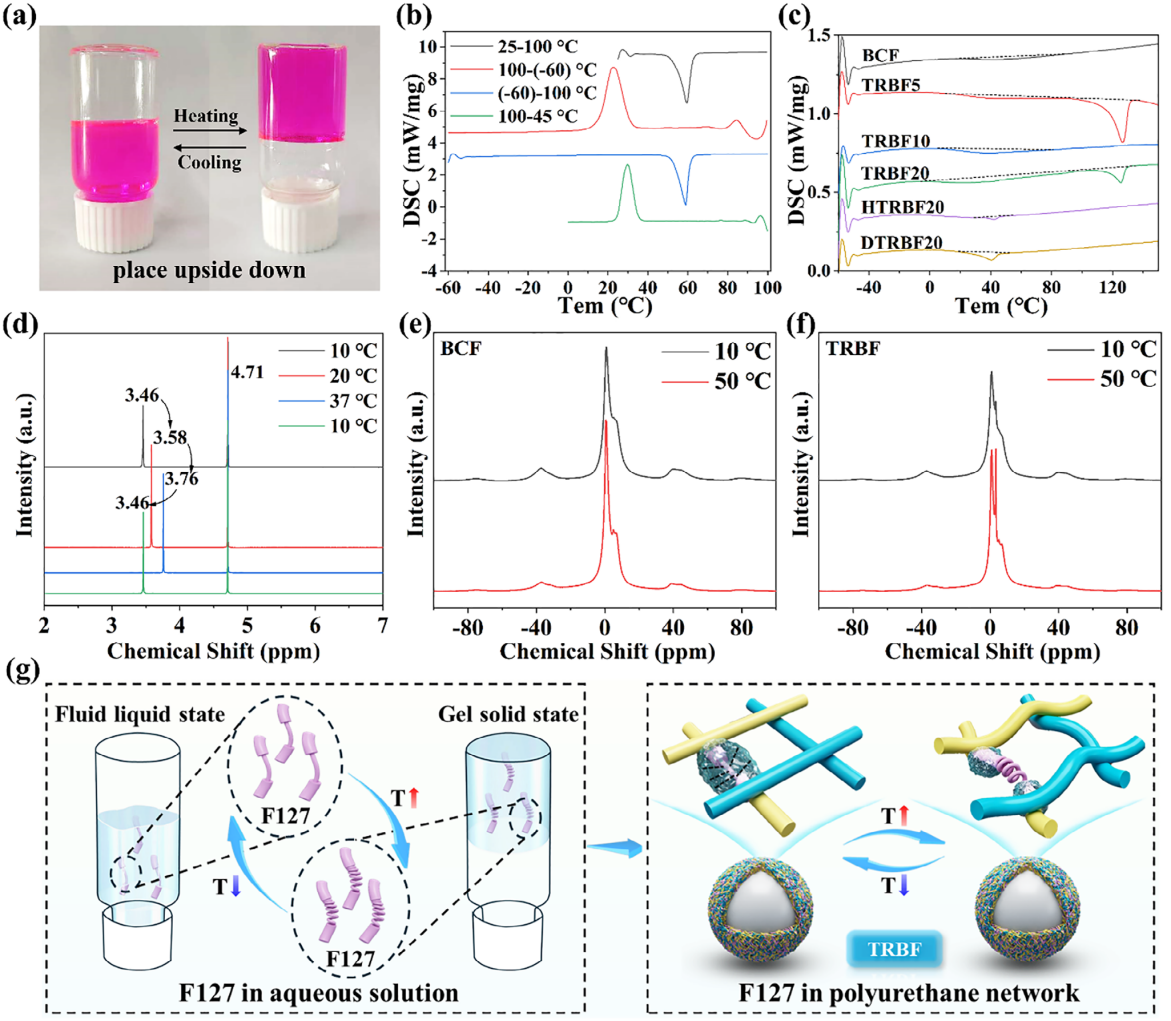
(a) Images of the inverted placement of F127 solution at different temperatures. DSC spectra of (b) F127 and (c) BCF, TRBF5, TRBF10, TRBF20, HTRBF20, and DTRBF20 (−60°C to 150°C). (d) NMR spectra of F127 at different temperatures. (e,f) Solid‐state NMR spectra of BCF and TRBF10 at different temperatures. (g) Schematic diagram of morphological evolution of F127 in aqueous solution and polyurethane matrix.

The ^1^H NMR spectra (Figure 5d–f) decode the temperature‐responsive molecular events of F127 and its role in endowing castor oil‐based polyurethanes with dynamic segmental behavior at the molecular scale. F127 was dissolved by D_2_O (Figure 5d), and the shift in the poly(propylene oxide) (PPO) segment signal (3.46 ppm at 10°C, 3.58 ppm at 20°C, and 3.76 ppm at 37°C) originated from the micellization behavior of F127: below the critical micelle temperature (CMT), PPO was fully solvated in the polar aqueous environment; above the CMT, the hydrophobic PPO segments aggregated into a nonpolar micellar core, reducing proton shielding and causing a shift to lower fields. This confirms that F127 is a temperature‐switchable building block with environmentally responsive PPO domains.

For BCF (Figure 5e), the broad ^1^H signals at 10°C and 50°C showed negligible temperature‐dependent variation. This indicates that the densely crosslinked network and persistent hydrogen‐bonding interactions of BCF, which rigidify the matrix and suppress segmental mobility, even moderate heating (to 50°C) fail to relax these constraints. In contrast, the TRBF (Figure 5f) incorporating F127 exhibited pronounced temperature responsiveness: at 10°C, a weak signal at ∼3.5 ppm (matching F127's PPO segment in Figure 5d) appeared, corresponding to PPO domains confined within the rigid network of the TRBF (limited motion yields faint signals). At 50°C, the signal caused by PPO was significantly enhanced: the increase in temperature triggered the hydrophobic aggregation of PPO (recapitulating its solution behavior in Figure 5d), which relaxed local crosslinking constraints and enhanced the segmental mobility of PPO, improving nuclear spin relaxation and amplifying signal intensity. This divergence from BCF demonstrates that F127 introduces temperature‐responsive dynamic domains into the polyurethane matrix, effectively combining the phase thermosensitivity of F127 with the segmental kinetics of the bulk network.

XPS was used to probe the elemental composition and valence states of BCF and TRBF10 at 4°C and 50°C (Figure 6a). The characteristic peaks of C 1s (284.4 eV), N 1s (399.8 eV), and O 1s (532.0 eV) remained unchanged at both temperatures [58, 59]. Peak fitting (Figure S7) confirmed that all surface chemical states were stable upon heating, consistent with in situ FTIR results (Figure S8), which showed no spectral changes from 10°C to 50°C. These findings indicate that both materials maintain stable chemical structures during heating.

**FIGURE 6:**
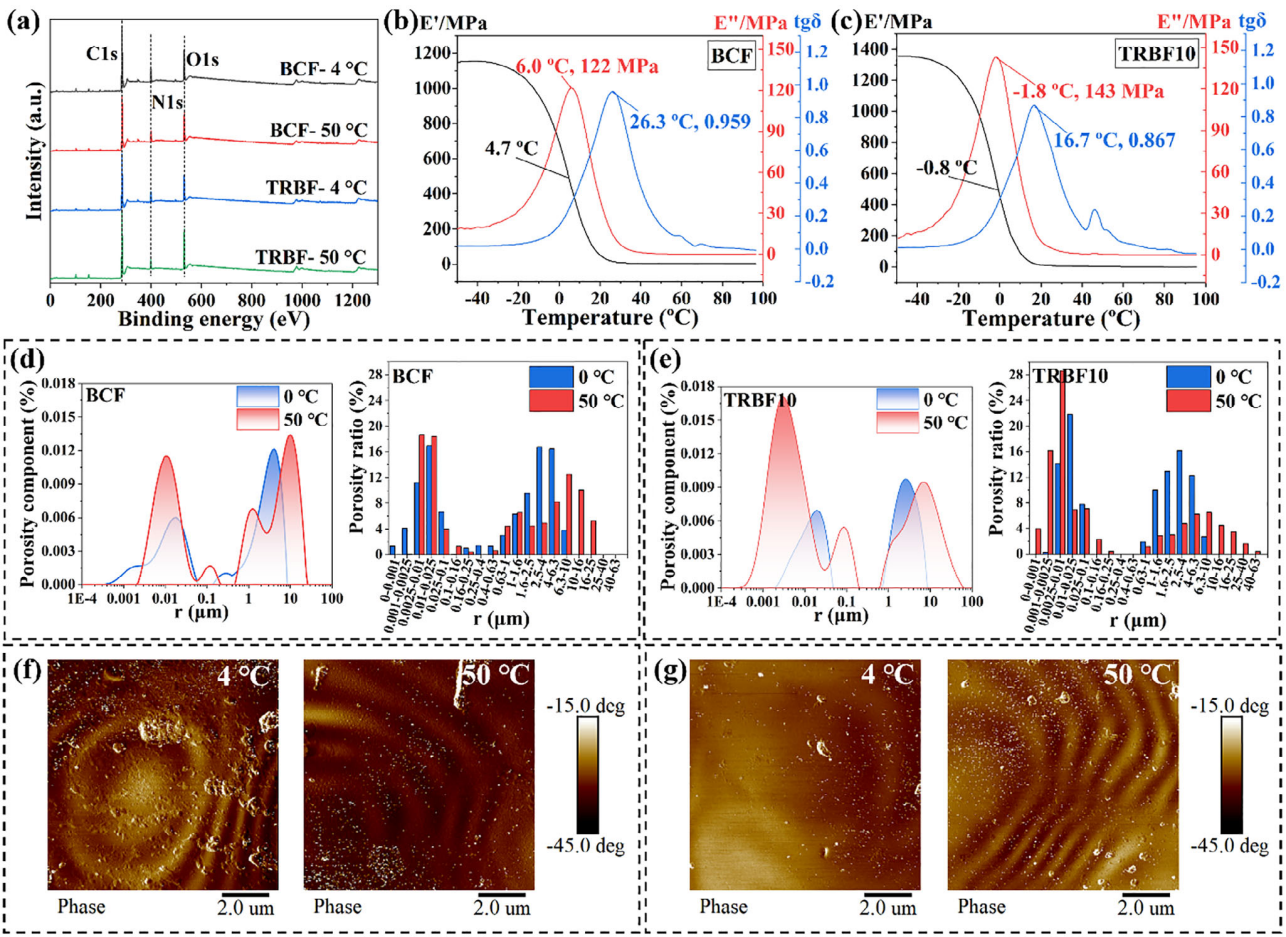
(a) XPS survey spectra of BCF and TRBF10 at different temperatures. (b,c) DMA curves (storage modulus, loss modulus, and tan δ) for BCF and TRBF10. The porosity component diagrams (left) and pore radius distribution histograms (right) of (d) BCF and (e) TRBF10 at different temperatures. Note that the x‐axis of the porosity component diagrams is plotted on a logarithmic scale to visualize the distribution of pores spanning multiple orders of magnitude (0.0001–100 µm). AFM phase images of BCF (f) and TRBF10 (g) at different temperatures.

DMA curves were analyzed to evaluate viscoelastic behavior and chain dynamics (Figure 6b,c). The storage modulus (*E′*) of both materials decreased with increasing temperature, indicating that the elasticity of the material gradually decreases. TRBF10 exhibited a lower modulus inflection point (−0.8°C) than BCF (4.7°C) and a steeper modulus decline. This is attributed to the flexibility introduced by the F127 block copolymer, which alters interchain interactions and crystallization behavior of the material. TRBF10 also showed a higher loss modulus (E″ = 143 MP) than that of BCF (122 MPa), indicating greater viscosity. At 20°C, however, both *E*′ and *E*′′ of TRBF10 were significantly reduced, suggesting a more favorable molecular chain arrangement. This structural change facilitates molecular transport and accelerates nutrient release.

The crosslinking density (*ν_e_*) was further calculated from the rubber plateau modulus using rubber elasticity theory. The *ν_e_* ​ of TRBF10 (235.08 mol/m^3^) was significantly higher than that of BCF (128.62 mol/m^3^), which provides structural support for its superior controlled‐release performance.

The loss tangent (*tag δ*) reflects the glass transition temperature (*Tg*) of the material. The *Tg* of BCF was 26.3°C, whereas that of TRBF10 was 16.7°C, indicating that the incorporation of F127 effectively lowers the *Tg* of polyurethane. The crosslinking density (*ν_e_*) was further calculated from the rubber plateau modulus using rubber elasticity dynamics theory. The *ν_e_* of TRBF10 (235.08 mol/m^3^) was significantly higher than that of BCF (128.62 mol/m^3^), which provides structural support for its superior controlled‐release performance [60, 61].

$$E^{\prime }=3v_{e}R$$

where *T* is the absolute temperature at (*Tg*+40)°C, *E′* is the storage modulus at (*Tg*+40)°C, and *R* is the gas constant.

Porosity is a dominant factor governing the controlled‐release performance of the material. To evaluate the temperature effect on coating porosity, low‐field NMR relaxometry was employed to determine the porosities of BCF and TRBF10 at 0°C and 50°C (Figure 6d,e). At 0°C, the porosities of BCF and TRBF10 were 0.40% and 0.35%, respectively. Upon heating to 50°C, these values increased to 0.60% and 0.85%, corresponding to a 2.43‐fold higher thermal responsiveness for TRBF10. This enhanced sensitivity stems from a unique temperature‐activated mechanism. As shown in the pore size distribution histograms and cumulative curves (Figure 6 and Figure S9), BCF exhibits a slight shift of existing pores toward larger radii due to thermal expansion, without generating new pores. In contrast, the TRBF not only shifts preexisting pores to larger sizes but also creates a new population of detectable pores centered at ∼0.001 µm, arising from thermally induced molecular chain contraction (Figure 6e). Nanoscale pore unlocking accounts for the substantial porosity increase and underpins the superior thermal responsiveness of TRBFs.

AFM phase images further confirmed the microstructural differences (Figure 6f–g). At 4°C, BCF showed uniform mixing of hard and soft segments with minimal microphase separation, and its microstructure remained nearly unchanged at 50°C, indicating low temperature sensitivity. In contrast, TRBF10 exhibited clear phase contrast at 4°C with numerous bright spots, likely representing micelles formed by F127. At 50°C, alternating light and dark bands were observed, indicating maintained microphase separation and localized aggregation. These results demonstrate that the incorporation of F127 significantly enhances the temperature responsiveness of the coating.

The WVP coefficient differed significantly among samples (P = 0.003, one‐way ANOVA) at 38°C. TRBF5 (2.22 ± 0.07) maintained a low permeability comparable to that of BCF (2.00 ± 1.27), whereas higher F127 addition increased the WVP coefficient to 5.05 ± 0.46 for TRBF10 and 7.83 ± 0.86 for TRBF20 (Figure 7b). Overall, the WVP coefficient followed the order of BCF < TRBF5 < TRBF10 < TRBF20, which is consistent with the nutrient‐release behavior observed at 38°C (Figure 4 and Figure S3), indicating that the enhanced nutrient release is closely associated with the increased water vapor permeability of the polyurethane coating. MD simulations were performed to verify the temperature‐responsive controlled‐release behavior of BCF and TRBF10 (Figure 7c–i). The total energy profiles of both BCF and TRBF10 systems exhibited smooth energy relaxation, indicating that the systems were stable and reached thermodynamic equilibrium, thus providing a reliable basis for subsequent structural analyses. The radius of gyration (*Rg*) analysis shows that the CO component in both systems remains conformationally stable under heating. In contrast, the *Rg*​ of F127 in TRBF10 gradually increased, indicating that the F127 chains underwent a conformational transition to a more extended state under the combined effects of thermal activation and hydration. Correspondingly, the number of water‐polymer hydrogen bonds in TRBF10 increased significantly with temperature, while that in BCF remained nearly constant. Similarly, the fractional free volume (FFV) of TRBF10 exhibited a clear temperature‐dependent increase, whereas BCF showed negligible variation. These observations suggest that the incorporation of F127 enhances the hydrophilicity of TRBF10, thereby facilitating water penetration into the polymer matrix. Additionally, the increased FFV in TRBF10 illustrates enhanced void content and connectivity with rising temperature and provides additional pathways for water diffusion, which is consistent with the hydrogen bond analysis. Snapshots of molecular transport trajectories in the polymer‐water interfacial models at different temperatures visually confirmed these observations, showing more pronounced water penetration into the TRBF10 matrix compared to BCF under heating conditions. These structural features synergistically facilitate water penetration, which subsequently promotes temperature‐modulated nutrient diffusion through the coating and explains the experimentally observed release behavior.

**FIGURE 7:**
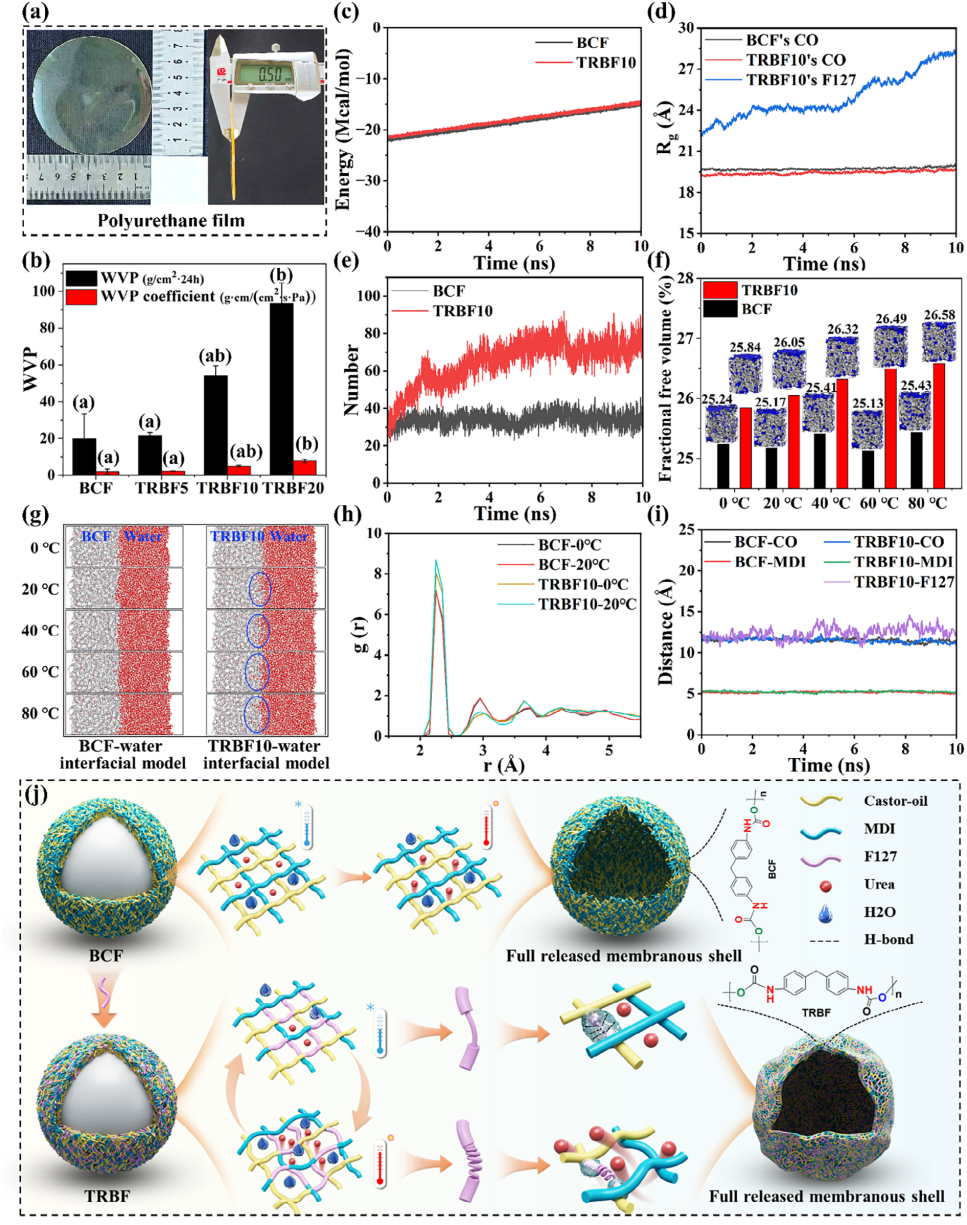
(a) Digital photograph of the circular polyurethane film specimen used for the WVP test (diameter: 75 ± 1 mm; thickness: 0.50 ± 0.03 mm) and (b) WVP results of BCF and TRBFs. Data are means ± standard deviation (*n* = 3). One‐way ANOVA was performed, followed by Tamhane's T2 post‐hoc test for multiple comparisons due to unequal variance. Different lowercase letters denote significant differences at *p*< 0.05. (c) The total energy profiles and (d) Polymer radius of gyration of both BCF and TRBF10 systems during the 10 ns MD heating simulation. (e) The hydrogen bond changes in the heating polymer‐water interface models. (f) Fractional free volume (FFV) and (g) Snapshots of molecular transport trajectories in the polymer‐water interface models at various temperatures. The 3D visualization inserts show the spatial distribution of free volume cavities (blue) within the polymer matrix (gray). (h) The O─O radial distribution function and (i) the end‐to‐end distance in the heated polymer‐water interface models. (j) Mechanism illustrating how temperature‐induced structural changes regulate water penetration and subsequent nutrient release in TRBF.

The O─O radial distribution function (RDF) was employed to characterize interfacial hydrogen bonding. At ∼2.3 Å, corresponding to hydrogen bonding at the polymer‐water interface, TRBF10 exhibited a higher peak intensity than BCF, which further increased with rising temperature, whereas BCF showed no obvious temperature dependence. This indicates stronger interfacial hydrogen bonding in TRBF10, which is further enhanced at elevated temperatures. These results demonstrate that the incorporation of F127 promotes water ordering and hydrogen bonding, improving interfacial hydrophilicity at high temperatures and thereby facilitating water penetration and nutrient release (Figure 7h). In contrast, the C‐H RDFs of both BCF and TRBF10 remained unchanged, confirming that the polymer backbone remained structurally intact throughout the simulations. The density profiles further revealed that the segments were not randomly distributed but exhibited layered structures and microphase separation (Figure S10).

The end‐to‐end distance (Ree) was used to evaluate the conformational stability and mobility of different segments. The Ree values of CO and MDI segments remained nearly constant in both systems, indicating stable conformations and low mobility of the polyurethane matrix components. In contrast, F127 chains in TRBF10 exhibited significantly larger fluctuations in Ree, reflecting higher chain mobility and conformational flexibility, with pronounced dynamic contraction‐extension behavior. This inherent dynamic characteristic provides a structural basis for the temperature‐responsive performance of TRBF10 (Figure 7i).

In summary, BCF and TRBF10 exhibit distinct mechanisms underlying their controlled‐release behavior (Figure 7j). For BCF, nutrient release is primarily governed by temperature‐independent diffusion through the polyurethane coating. Although increasing temperature slightly accelerates molecular motion, the diffusion pathway remains essentially unchanged, resulting in a conventional diffusion‐controlled release behavior. In contrast, the incorporation of F127 endows TRBF with a temperature‐responsive coating structure. At low temperatures, hydrated F127 domains together with the compact polyurethane network suppress water penetration, thereby maintaining a low nutrient‐release rate. As the temperature increases above the phase‐transition temperature of F127, dehydration and conformational rearrangement of the F127 segments increase the free volume and pore connectivity of the coating, as supported by the porosity analysis, WVP measurements, and MD simulations. These structural changes facilitate water penetration into the coating and promote temperature‐modulated diffusion of nutrients, leading to accelerated nutrient release at elevated temperatures.

Notably, this work represents an early‐stage proof‐of‐concept of F127‐modified biobased polyurethane coatings. Among the tested F127 loadings (5, 10 and 20 wt.%), TRBF10 strikes the optimal balance between nutrient retention and thermoresponsiveness; lower loading yields weak temperature sensitivity, while excessive loading breaks the crosslinked network and abolishes controlled release. Simply adjusting F127 dosage offers only limited ability to tune the phase transition temperature. Developing a suite of formulations with tailored response windows for global climatic and cropping diversity remains a long‐term goal, requiring molecular modification of thermosensitive moieties or alternative responsive components rather than mere dosage adjustment. This foundational feasibility study of the F127–castor oil polyurethane system provides a structural basis for future graded thermoresponsive fertilizer development.

### 3.6 Scalability and Economic Considerations

From a translational perspective, the practical application of TRBFs relies on both scalable manufacturing and economic viability. The rotary drum‐coating process used in this work is fully compatible with existing commercial CRF production lines, and all raw materials are commercially available at industrial scale. The low optimal F127 loading (0.3 wt.% of the final fertilizer product) further minimizes the additional material requirement. A comparison of different fertilizer systems is summarized in Table S2. Although a comprehensive techno‐economic analysis is beyond the scope of this laboratory‐scale study, the compatibility of the present formulation with existing manufacturing processes provides a solid foundation for future scale‐up. Future work will focus on detailed techno‐economic analysis, multi‐site field trials, and cradle‐to‐field life cycle assessment to comprehensively evaluate the economic and environmental performance of TRBFs.

## 4 Conclusion

This study pioneers the in situ incorporation of a thermosensitive block copolymer F127 (PEG‐PPG‐PEG) into a polyurethane network derived from biobased castor oil and MDI, achieving temperature‐responsive coatings for CRFs. The incorporation of F127 does not alter the spatial structure or thermal stability of the original polyurethane but increases the elasticity of the coatings and enhances the microphase separation behavior. F127 replaces part of the castor oil in the coating material, enabling the coating to respond to temperature changes and modulating its controlled‐release properties. TRBF10 exhibits a density of 1.21 g/cm^3^ and a crosslink density of 235.08 mol/m^3^, accompanied by excellent temperature‐controlled release performance: nutrient release is suppressed to only 9.22% over 30 days at 10°C, but sharply accelerates to 80.32% at 30°C. This remarkable switching behavior arises mainly from thermally induced porosity evolution: the porosity of TRBF increases from 0.35% to 0.85% with rising temperature, significantly exceeding that of BCF (0.40%–0.60%). Molecular dynamics simulations further confirm that thermal expansion of F127 chains increases free volume and enhances hydrogen bonding, thus facilitating water penetration and diffusion. This intelligent response pattern overcomes the “single‐rate” bottleneck of traditional diffusion‐controlled release, providing valuable insights for the design of precise, efficient, and eco‐friendly next‐generation smart CRFs and advancing the development of sustainable controlled‐release materials.

## Author Contributions

**Jiahui Liu**: writing –original draft, investigation, validation, methodology, data curation, software. **Junyin Li**: methodology, validation. **Ziyao Li**: validation, investigation. **Xiangjie Zhao**: methodology, investigation. **Fengyan Fang**: investigation, validation. **Yan Liu**: data curation, investigation. **Songyuan Wang**: validation, investigation. **Boning Su**: software, validation. **Yuanyuan Yao**: supervision, formal analysis, visualization. **Bin Gao**: writing –review and editing. **Shugang Zhang**: project administration, supervision, writing –review and editing. **Yuechao Yang**: conceptualization, resources, project administration, funding acquisition.

## Conflicts of Interest

The authors declare no conflicts of interest.

## Supporting information




**Supporting File**: advs78371‐sup‐0001‐SuppMat.docx.

## Data Availability

The data that support the findings of this study are available from the corresponding author upon reasonable request.
