# Supplementary material for: Poloxamer‐Enabled Thermoresponsive Biobased Controlled‐Release Fertilizer for On‐Demand Nutrient Delivery
Source: Adv Sci (Weinh). 2026 Oct 18:e78371. Online ahead of print. doi: 10.1002/advs.78371 (PMC13676992; doi:10.1002/advs.78371)
Supplement: Supplementary file 1 [file ADVS-9999-e78371-s001.docx]

Poloxamer-Enabled Thermoresponsive Biobased Controlled-Release Fertilizer for On-Demand Nutrient Delivery

Jiahui Liu ^a^, Junyin Li ^a^, Ziyao Li ^a^, Xiangjie Zhao ^a^, Fengyan Fang ^a^, Yan Liu ^a^, Songyuan Wang ^a^, Boning Su ^a^, Yuanyuan Yao ^a^, Bin Gao ^b^, Shugang Zhang ^a^ *, Yuechao Yang ^a, c, d^ *

^a^ National Engineering Research Center for Efficient Utilization of Soil and Fertilizer Resources, National Engineering & Technology Research Center for Slow and Controlled Release Fertilizers, College of Resources and Environment, Shandong Agricultural University, Taian, Shandong 271000, China

^b^ Department of Civil and Environmental Engineering, Rensselaer Polytechnic Institute, Troy, NY 12180, USA

^c^ State Key Laboratory of Wheat Improvement, Shandong Agricultural University, Taian 271000, China

^d^ Department of Soil and Water Sciences, Tropical Research and Education Center, Institute of Food and Agricultural Sciences, University of Florida, Homestead, FL, 33031, USA

*Corresponding authors

Shugang Zhang, E-mail: shugangzhang2014@163.com

Yuechao Yang, E-mail: yangyuechao2010@163.com

**Table S1.** Formulation compositions of BCF and TRBFs coating materials.

| Sample | F127 (g) | CO (g) | MDI (g) | Acetone (g) | F127 content (wt%) | Urea (g) | Coating rate (%) |
| --- | --- | --- | --- | --- | --- | --- | --- |
| BCF | 0 | 14.4 | 9.6 | 6 | 0 | 800 | 3 |
| TRBF5 | 1.2 | 13.68 | 9.12 | 6 | 5 | 800 | 3 |
| TRBF10 | 2.4 | 12.96 | 8.64 | 7.8 | 10 | 800 | 3 |
| TRBF20 | 4.8 | 11.52 | 7.68 | 9 | 20 | 800 | 3 |
| HTRBF20 | 4.8 | 11.52 | 7.68 | 9 | 20 | 800 | 3 |

Note: Each coating cycle employed 0.5 wt % of the total urea mass. After 6 consecutive cycles, the coating rate reaches 3%.

**F****ig. S1** (a) Schematic diagram of fabrication process and (b) Molecular structure synthesis mechanism of BCFs and TRBFs.

**Fig. S2** SEM images with 10000 × magnification of the (a) outer and (c) inner of BCF. SEM images with 10000 × magnification of the (b) outer and (d) inner of TRBF10. Microscopic image of (e) BCF film and (f) TRBF10 film.

**Fig. S3** (a-d) The cumulative nitrogen release rates of BCF, TRBF5, TRBF10, TRBF20, HTRBF20 and DTRBF20 at different temperatures. (e-l) The phase nitrogen release rates of BCF, TRBF5, TRBF10, TRBF20, HTRBF20 and DTRBF20 at different temperatures.

Figure S3a‑d presents cumulative nitrogen release rates (CNRs) of BCF, TRBF5, TRBF10, TRBF20, HTRBF20 and DTRBF20 incubated at 4 °C, 10 °C, 25 °C and 35 °C. For all samples, nitrogen release accelerated as incubation temperature increased. The reference BCF exhibited slow, temperature‑insensitive diffusional release across the whole temperature range. By contrast, TRBF series displayed obvious temperature‑dependent release behavior. At low temperatures (4 °C and 10 °C), TRBFs maintained suppressed nitrogen release; when temperature rose to 25 °C and 35 °C, nutrient release was greatly promoted. Among formulations, HTRBF20 and DTRBF20 achieved the highest cumulative nitrogen release at elevated temperatures.

Figure S3e‑l shows the phase nitrogen release rates (SNRs) at temperatures ranging from -4 °C to 35 °C. Sharp SNR peaks were observed for thermoresponsive TRBFs, and the peak position shifted toward shorter incubation time with increasing temperature. Such SNR peaks reflect the thermally triggered “switch‑on” nutrient‑release event of poloxamer‑modified polyurethane coating. The non‑responsive BCF only maintained low and flat SNR values without prominent release peaks, further confirming its passive diffusion‑dominated release mechanism. The complete release dataset is provided in the dataset (Release Rate Dataset).

**Fig. S4** The physical photos of BCF, TRBF5, TRBF10, TRBF20, HTRBF20 and DTRBF20 on the 1st, 3rd, 7th and 21st days of cultivation.

**Fig. S5** The factor-effect relationship diagram and 3D response surface construction model of (a) TRBF5, (b) TRBF20, (c) HTRBF20 and (d) DTRBF20.

**Fig. S6** DSC spectra of BCF, TRBF5, TRBF10, TRBF20, HTRBF20 and DTRBF20 (25–150 ℃).

**Fig. S7** XPS peak-fitting curves of characteristic elements of (a-b) BCF and (c-d) TRBF.

The C-C/C-H (283.8 eV), C-O/C-N (285.3 eV), and O-C=O (288.0 eV) shown in the C1s spectrum of XPS are the characteristic chemical bonds of polyurethane. The C-N (399.2 eV) bond in the N1s fitting curve proves the successful preparation of polyurethane, and -NCO (400.2 eV) in Fig. S7 is a small amount of reactive residual MDI. The large difference between the peak areas of C=O (532.4 eV) and C-O (533.2 eV) in the O1s fitting curve may be due to the addition of F127.

**Fig. S8** In-situ FTIR of (a) BCF, (b) TRBF10, (c) TRBF20 and (d) HTRBF.

**Fig. S9** Cumulative pore radius distribution diagrams of (a) BCF and (b) TRBF10 at different temperatures.

**Fig. S10** (a) The C-H radial distribution function in the heating polymer-water interface models. The density profiles of (b-c) BCF and (d-e) TRBF10 system during the 10 ns MD heating simulation.

**Table S2.** Comparison of Bio-based nitrogen fertilizers and thermoresponsive controlled-release fertilizer systems for agricultural applications.

| **Parameter** | Bio-based controlled release fertilizer (BCF) | Thermoresponsive fertilizer (TRBF10) | Future considerations |
| --- | --- | --- | --- |
| Nitrogen release mechanism | Passive diffusion-controlled release through coating | Environment-responsive release regulated by temperature | Optimization of responsiveness under field conditions |
| Response to environmental variation | Limited; mainly determined by coating properties | Adaptive response to temperature fluctuations | Validation under diverse climates and soils |
| Synchronization with crop nutrient demand | Moderate | Potentially improved due to environmental responsiveness | Long-term field evaluation required |
| Nitrogen loss risk | Reduced compared with urea | Expected to be further reduced through adaptive release behavior | Quantification by field-scale nitrogen balance studies |
| Fertilization efficiency | Improved | Potentially enhanced | Require agronomic performance evaluation |
| 3 wt.% coating‑material consumption | 30 kg ton^-1^ fertilizer | 30 kg ton^-1^ fertilizer (containing 3 kg F127) | Cost reduction through large-scale synthesis |
| Manufacturing complexity | Fully mature industrial production | Fully mature fluidized-bed coating process | Process optimization and scale-up production |

**Seed Germination Assay**

Surface-sterilized winter wheat seeds were subjected to a germination assay under four temperature regimes (15, 20, 25, and 30 °C) with four treatments: blank control (CK, no fertilizer), pure urea granulesD (U), conventional coated controlled-release fertilizer (BCF), and poloxamer-modified thermoresponsive controlled-release fertilizer TRBF10 (TR). The experiment followed a completely randomized design with three biological replicates per treatment. Briefly, 15 sterilized seeds were sown in 200 mL cups filled with 200 g washed quartz sand; one fertilizer granule (0.04 ± 0.02 g) was embedded in the sand matrix for each fertilized group. All cups were initially supplied with 60 mL sterile water and maintained at constant moisture by daily water replenishment in growth chambers under a 16 h light / 8 h dark photoperiod. Seeds with radicle protrusion ≥ 1 mm were counted as germinated, and daily counting was performed for 15 consecutive days. Final germination percentage and germination index (GI) were calculated accordingly.

**Fig. S11** Germination responses of winter wheat seeds under different temperature and fertilizer treatments. (a–d) Dynamic cumulative germination curves of wheat seeds under different temperature regimes (15, 20, 25, and 30 °C), respectively. (e) Final germination percentage and (f) germination index (GI) after 15 days of incubation. Individual replicate values are shown as open symbols, and bars represent mean ± SD (n = 3). (g) Representative photographs of wheat seedlings after 15 days of germination. CK, blank control without fertilizer; U, pure urea; BCF, conventional coated controlled-release fertilizer; TR, thermoresponsive TRBF10 fertilizer.

**Table S3.** Two-way analysis of variance (Type III sum of squares) for final germination percentage of wheat seeds as affected by temperature and fertilizer treatment.

| **Source** | **Type III Sum of Squares** | **df** | **Mean Square** | ***F* value** | ***P* value** | **Partial η²** | **Remark** |
| --- | --- | --- | --- | --- | --- | --- | --- |
| Corrected Model | 15281.472 | 15 | 1018.765 | 3.705 | 0.001 | 0.635 | ** |
| Intercept | 44409.550 | 1 | 44409.550 | 161.485 | <0.001 | 0.835 | ** |
| Temperature | 9951.380 | 3 | 3317.127 | 12.062 | <0.001 | 0.531 | ** |
| Treatment | 869.594 | 3 | 289.865 | 1.054 | 0.382 | 0.090 | ns |
| Temperature × Treatment | 4460.498 | 9 | 495.611 | 1.802 | 0.107 | 0.336 | ns† |
| Error | 8800.222 | 32 | 275.007 | — | — | — |  |
| Total | 68491.245 | 48 | — | — | — | — |  |
| Corrected Total | 24081.695 | 47 | — | — | — | — |  |

Note: Type III sums of squares were used for two-way ANOVA. ns, not significant (*P* > 0.05); ** *P* < 0.01; † indicates a marginal trend (0.05 < *P* < 0.10). Partial η² represents effect size. The model explained 63.5% of the variance (R² = 0.635; adjusted R² = 0.463). Levene’s test indicated significant heterogeneity of variance among groups (*F* (15,32) = 3.580, *P* = 0.001). Therefore, Tamhane’s T2 test was applied for post hoc comparisons because it does not assume equal variances.

To evaluate whether the thermoresponsive fertilizer could translate its temperature-dependent release behavior into biological performance, wheat germination assays were conducted under four temperature regimes (15, 20, 25, and 30 °C) with different nitrogen sources.

Due to significant heterogeneity of variance among groups (Levene’s test, P = 0.001), Type III two-way ANOVA was performed considering the balanced experimental design, and Tamhane’s T2 test was used for post hoc comparisons.

Temperature exerted a highly significant effect on the final germination percentage (F = 12.062, P < 0.001, partial η² = 0.531), indicating that environmental temperature was the dominant factor determining wheat germination performance. Germination was strongly inhibited at 30 °C, consistent with the physiological preference of wheat as a cool-season crop.

The main effect of fertilizer treatment was not significant (F = 1.054, P = 0.382), suggesting that external nitrogen sources had limited influence on final germination percentage during the early germination stage. This result is reasonable because seed germination is mainly supported by endogenous nutrient reserves stored in the endosperm, while external fertilizers primarily affect subsequent seedling establishment and nutrient acquisition.

Although the interaction between temperature and fertilizer treatment was not statistically significant (F = 1.802, P = 0.107), the relatively large effect size (partial η² = 0.336) suggested a temperature-dependent tendency in fertilizer responses. In particular, the thermoresponsive TR treatment displayed a distinct response pattern across temperature gradients, which was consistent with its temperature-triggered nitrogen release mechanism.

**Table S4.** Two-way analysis of variance (Type III sum of squares) for germination index (GI) of wheat seeds as affected by temperature and fertilizer treatment.

| **Source** | **Type III Sum of Squares** | **df** | **Mean Square** | ***F* value** | ***P* value** | **Partial η²** | **Remark** |
| --- | --- | --- | --- | --- | --- | --- | --- |
| Corrected Model | 8.275 | 15 | 0.552 | 3.158 | 0.003 | 0.597 | ** |
| Intercept | 0.670 | 1 | 0.670 | 3.837 | 0.059 | 0.107 | ns |
| Temperature | 4.731 | 3 | 1.577 | 9.028 | <0.001 | 0.458 | ** |
| Treatment | 0.360 | 3 | 0.120 | 0.687 | 0.567 | 0.061 | ns |
| Temperature × Treatment | 3.184 | 9 | 0.354 | 2.025 | 0.069 | 0.363 | ns† |
| Error | 5.590 | 32 | 0.175 | — | — | — |  |
| Total | 14.535 | 48 | — | — | — | — |  |
| Corrected Total | 13.865 | 47 | — | — | — | — |  |

Note: Type III sums of squares were used for two-way ANOVA. ns, not significant (P > 0.05); ** P< 0.01; † indicates a trend toward significance (0.05 < P < 0.10). Partial η² represents the effect size. The model explained 62.3% of the total variance (R² = 0.623; adjusted R² = 0.447). Levene’s test indicated no significant heterogeneity of variance among groups (F(15,32) = 0.691, P = 0.774). Tamhane’s T2 test was used for post-hoc pairwise comparisons (α = 0.05).

In addition to the final germination percentage, the germination index (GI) was further analyzed to evaluate early germination vigor and the potential biological response of wheat seeds to different fertilizer formulations. Two-way ANOVA was performed to determine the effects of temperature, fertilizer treatment, and their interaction on GI values.

Temperature exhibited a highly significant effect on wheat GI (*F* = 9.028, *P* < 0.001, partial η² = 0.458), indicating that environmental temperature was the primary factor regulating early germination performance. In contrast, the main effect of fertilizer treatment was not statistically significant (*F* = 0.687, *P* = 0.567), suggesting that external nitrogen sources had limited influence on seed vigor during the germination stage, when seedling development mainly relies on endogenous nutrient reserves.

Notably, the interaction between temperature and fertilizer treatment showed a tendency toward significance (*F* = 2.025, *P* = 0.069, partial η² = 0.363), indicating that fertilizer performance varied depending on environmental temperature conditions. Among the tested formulations, the thermoresponsive controlled-release fertilizer (TR) exhibited relatively stable GI values across different temperature regimes, particularly maintaining higher germination vigor under low-temperature conditions (15 °C), where the TR treatment achieved the highest GI value (0.690) compared with CK, urea, and conventional controlled-release fertilizer treatments.

The temperature-dependent variation in GI response was consistent with the designed thermoresponsive release behavior of TR, suggesting that the material could regulate nitrogen availability according to environmental temperature fluctuations. Although the interaction effect did not reach conventional statistical significance, the relatively large effect size (partial η² = 0.363) indicates a biologically meaningful tendency, supporting the potential of TR as an environmentally adaptive fertilizer platform.
